# Bronchial artery embolization as a life-saving method in a case of massive hemoptysis secondary to a hemorrhagic bullous emphysema

**DOI:** 10.1016/j.radcr.2025.02.025

**Published:** 2025-03-15

**Authors:** Youssef Bouktib, Ayoub El Hajjami, Badr Boutakioute, Merieme Ouali Idrissi, Najat Cherifi Idrissi El Ganouni

**Affiliations:** a Diagnostic and Interventional Radiology, Mohammed VI University Hospital Center in Marrakech, Marrakech, Morocco; b Higher Education in Diagnostic and Interventional Radiology, Mohammed VI University Hospital Center in Marrakech, Marrakech, Morocco; c Higher Education and Head of the Department of Diagnostic and Interventional Radiology, Mohammed VI University Hospital Center in Marrakech, Marrakech, Morocco

**Keywords:** Bullous emphysema, Hemoptysis, Bronchial artery embolization, Hemorrhagic bullae, Interventional radiology

## Abstract

Bullous emphysema, often associated with COPD, can lead to severe complications like massive hemoptysis. The Bronchial artery embolization (BAE) has become a well-established and effective procedure for the management of hemoptysis, which is the expectoration of blood from the lower respiratory tract. First introduced in the 1970s, BAE has evolved significantly due to advancements in interventional radiology techniques and embolic materials. The success rate of BAE in controlling acute hemoptysis ranges from 70% to 90% in the literature. However, recurrence rates remain a challenge, with studies reporting recurrence in up to 20%-30% of cases within the first year, often due to incomplete embolization or disease progression. Repeat embolization is frequently required in these patients, highlighting the importance of close follow-up and management of the underlying disease.

This case report describes a 55-year-old patient with a history of pulmonary tuberculosis, chronic smoking, and advanced COPD who presented with significant hemoptysis due to a hemorrhagic emphysematous bulla. Due to the patient's fragile condition, surgical intervention was deemed too risky, and embolization was chosen as a less invasive alternative. The procedure successfully controlled the bleeding without complications. This case highlights the importance of bronchial artery embolization (BAE) as a life-saving intervention in cases of massive hemoptysis, particularly in patients unfit for surgery. While BAE provides an effective solution for acute bleeding, long-term management of COPD and close follow-up are essential to prevent recurrence. A multidisciplinary approach is crucial for optimal patient outcomes.

## Introduction

Bullous emphysema, a complication of COPD, typically presents with pneumothorax. However, in rare cases, fluid accumulation within the bullae can lead to life-threatening complications, such as massive hemoptysis. We report a rare case of a 55-year-old patient with a history of pulmonary tuberculosis, chronic smoking, and advanced COPD, who presented with significant hemoptysis due to a large cystic lesion inside an emphysematous bulla. Despite extensive investigations ruling out infectious and embolic causes, the patient's fragile condition made surgical intervention impossible. This case highlights the importance of a comprehensive approach, which includes a thorough patient history, careful imaging analysis, and collaboration among a multidisciplinary team to effectively manage complex COPD-related complications.

## Case report

**Fig. 1:**
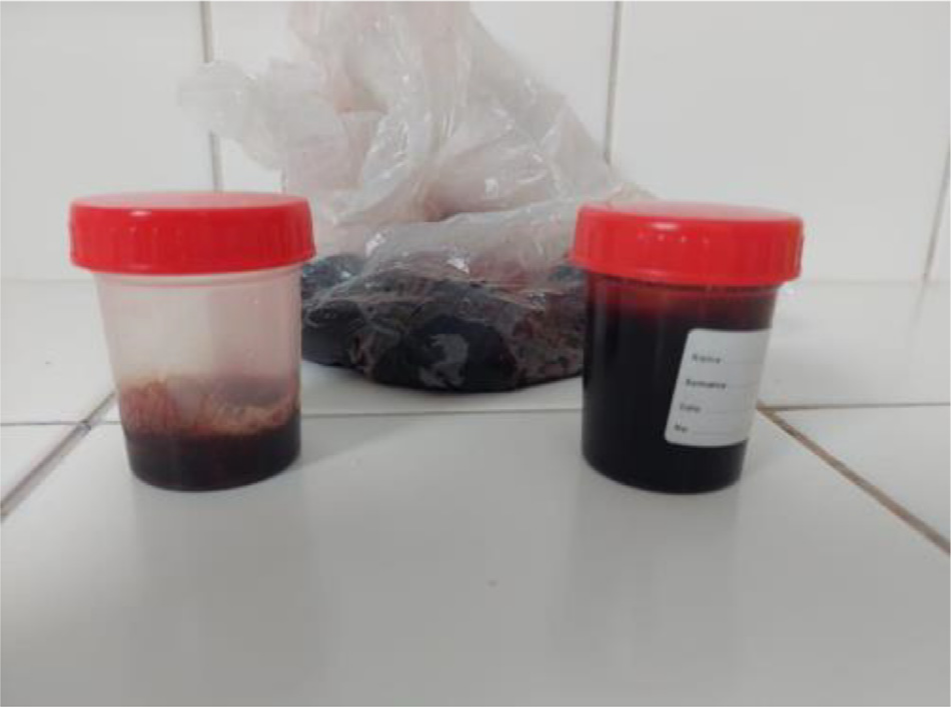
Large volume haemoptysis.

A 55-year-old patient with a history of pulmonary tuberculosis, chronic smoking, and COPD on dual bronchodilation therapy LABA (Long-Acting Beta-Agonist) and LAMA (Long-Acting Muscarinic Antagonist) presented with chronic hypercapnic respiratory failure managed with long-term oxygen therapy and home noninvasive ventilation. The patient also had a history of cor pulmonale, which was managed with diuretics to alleviate fluid overload and reduce right ventricular strain. The patient was admitted to the hospital due to worsening dyspnea and significant hemoptysis, without a history of trauma (Fig. 1). On physical examination, his oxygen saturation was 77% on room air, improving to 92% with 2L of oxygen via nasal cannula. He exhibited tachypnea at 25 breaths per minute and tachycardia at 105 beats per minute. Auscultation of the lungs revealed diminished breath sounds bilaterally, with no adventitious sounds such as rales, wheezing, or rhonchi detected. There was no evidence of stridor, and vocal resonance was unremarkable. The findings suggest reduced air entry, potentially indicative of underlying alveolar or pleural pathology

**Fig. 2:**
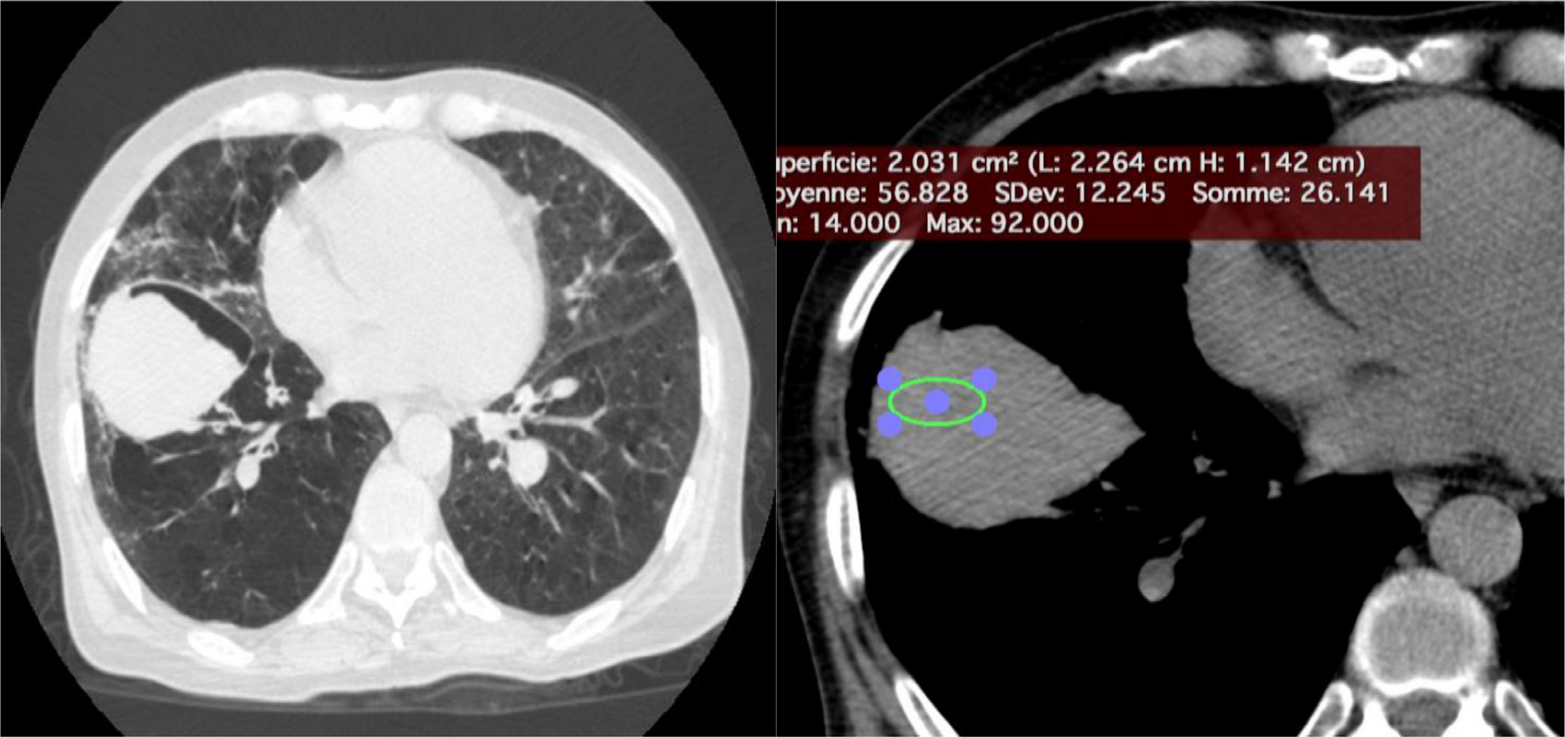
thoracic CT scan without and with contrast in mediastinal and parenchymal windows: emphysematous bulla in the middle lobe with hyperdense hemorrhagic content, noting the presence of surrounding ground-glass opacities corresponding to alveolar hemorrhage.

Laboratory results showed leukocytosis of 9,910 cells/μL, platelets at 233,000 cells/μL, a prothrombin time (PT) ratio of 100%, D-dimer levels of 360 ng/mL, and a C-reactive protein (CRP) of 16.5 mg/L. thoracic CT scan without and with contrast had demonstrated a emphysematous bulla in the middle lobe with hyperdense hemorrhagic content, noting the presence of surrounding ground-glass opacities corresponding to alveolar hemorrhage (Fig. 2). Bedside ultrasound revealed a heterogeneous, unvascularized cystic lesion on Doppler imaging with peripheral air-filled bullae.

A subsequent CT pulmonary angiogram (CT-PA) during admission revealed a large cystic structure measuring 73 × 58 × 107 mm, located in the lateral segment of the middle lobe, with an air-fluid level suggestive of a hemorrhagic bulla. No bleeding source or arteriovenous malformation was identified (Fig. 2).

Initial management included the intravenous administration of tranexamic acid at a dose of 1 gram every 8 hours and etamsylate at a dose of 500 mg every 6 hours to control hemoptysis resulting in a significant reduction in hemoptysis within five days of admission. The decision was made to pursue a less invasive approach with endovascular treatment, opting for systemic embolization.

**Fig. 3:**
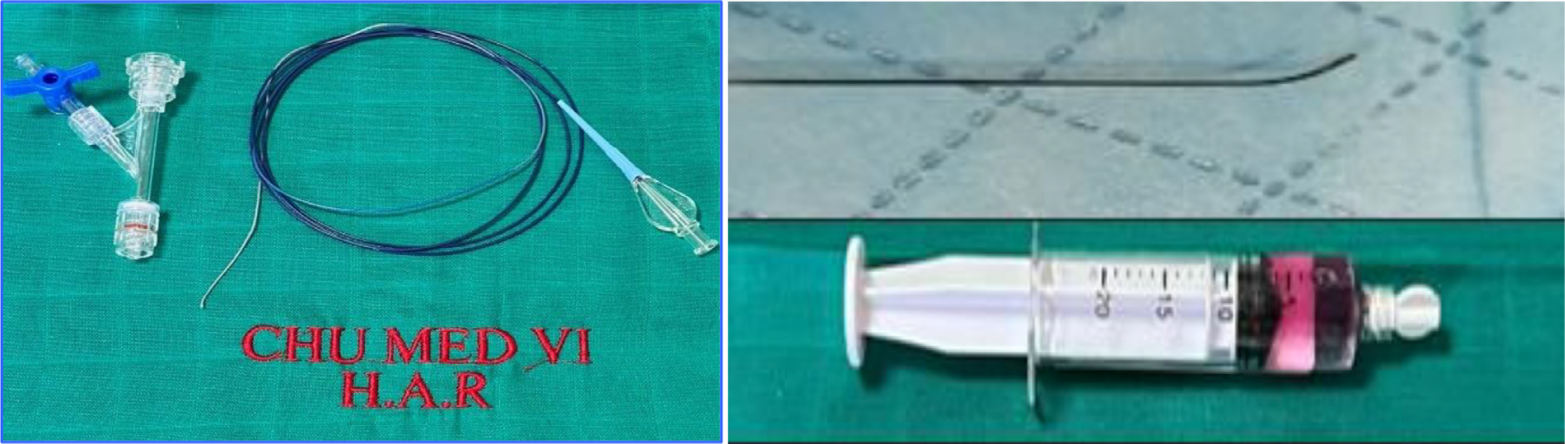
The materials used in the embolization procedure: Cobra C2 catheter, Progreat 2.9 Fr microcatheter with 800-micron microparticles.

**Fig. 4:**
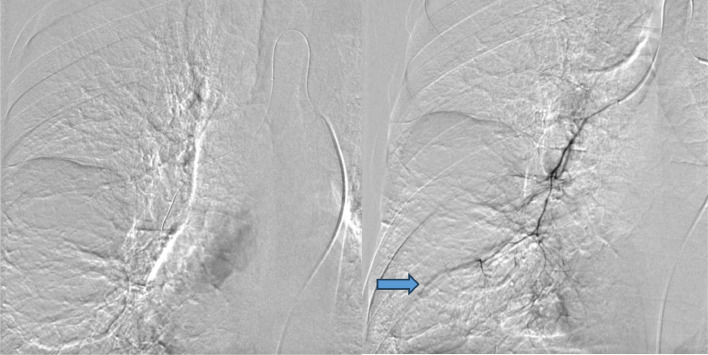
Arteriography images with subtraction showing selective catheterization of the right bronchial artery with identification of a distal vascular blush (arrow). Embolization with 800-um microparticles, flow reduction and disappearance of the blush.

The procedure was initiated via femoral vascular access using a 5F introducer, allowing the advancement of a Simmons-type navigation catheter. Selective catheterization of the right broncho-intercostal trunk revealed, upon contrast injection, a localized vascular blush in the middle lobe. Superselective catheterization of the right bronchial artery was then performed using a Progreat 2.9 Fr microcatheter, followed by embolization with 1 ml of 800 μm microparticles (Fig. 3) until a significant reduction in arterial flow was achieved. Postembolization angiographic control confirmed the complete resolution of the vascular blush (Fig. 4).

A follow-up appointment was scheduled 1 month after discharge, with no complications reported during the post-interventionnal period.".

## Discussion

The embolization of hemorrhagic emphysema bullae is a rare but effective treatment for patients experiencing severe complications, such as massive hemoptysis, typically seen in the context of advanced chronic obstructive pulmonary disease (COPD). Bullous emphysema, a hallmark of advanced COPD, can lead to life-threatening bleeding when bullae rupture or hemorrhage occurs. Managing such cases is particularly challenging due to the associated pulmonary dysfunction, multiple comorbidities, and the frailty of patients, which often preclude surgical interventions such as lobectomy.

In our case, embolization was selected as a less invasive alternative to surgery. This approach provided a short-term, effective solution for controlling the bleeding, avoiding the significant perioperative risks associated with lobectomy in patients with severe COPD. The decision to opt for embolization aligns with findings in the literature, which highlight that bronchial artery embolization (BAE) is a life-saving intervention in cases of massive hemoptysis, with success rates ranging from 70% to 90% in controlling acute bleeding episodes [1,2].

The principle of embolization in this context is to occlude the bleeding vessels, reducing blood flow to the hemorrhagic area and stopping the bleeding. Studies show that this targeted approach can be highly effective for patients who are not suitable candidates for surgery due to poor pulmonary function or other comorbidities [3,4]. Furthermore, embolization provides the added advantage of being repeatable if recurrent bleeding occurs, a common issue in patients with underlying structural lung diseases such as emphysema [5].

While embolization offers significant benefits, it is not without risks. The literature documents several potential complications, including chest pain, pulmonary infarction, and, in rare cases, inadvertent embolization of the spinal artery, which can result in paraplegia [6,7]. In our case, no major complications occurred, and the bleeding was successfully controlled, consistent with the outcomes seen in large case series and reviews of BAE for hemoptysisw management [8,9].

One important consideration is that while embolization addresses the acute issue of bleeding, it does not resolve the underlying disease process. Hemorrhagic emphysema bullae can recur, especially in patients who continue to smoke or have recurrent infections. Recurrence of hemoptysis has been reported in 10%-20% of cases within a few months after embolization, emphasizing the need for long-term follow-up and comprehensive management of the underlying COPD [10,11]. Preventive strategies, such as smoking cessation, optimal management of COPD, and regular monitoring with imaging, are crucial for reducing the risk of recurrence.

The literature also underscores the importance of a multidisciplinary approach in managing such complex cases. Collaboration between interventional radiologists, pulmonologists, and thoracic surgeons is vital to tailor treatment based on each patient's clinical status, imaging findings, and risk factors [12]. In particular, early involvement of interventional radiology is essential for timely decision-making and procedural planning, particularly when patients present with life-threatening hemoptysis [13].

In conclusion, the embolization of hemorrhagic bullous emphysema offers a safe and effective alternative to surgery in managing massive hemoptysis, particularly in high-risk patients. Although relatively rare, embolization has been well-documented as a crucial intervention in stabilizing patients with advanced COPD who are not surgical candidates. However, the underlying disease process requires continued attention, and a multidisciplinary approach is essential for improving long-term outcomes. Future studies with larger patient cohorts and longer follow-up will be invaluable in refining embolization techniques and improving patient care protocols.

## Patient consent

The patient provided informed consent for the publication of this case, including relevant medical details and accompanying images. The patient was fully informed about the embolization procedure for the hemorrhagic bulla, including its potential risks and benefits. The patient has agreed to share their medical information for the purposes of this scientific article, with the understanding that all identifying details have been anonymized to ensure confidentiality.
